# Effects of Mineralocorticoid Receptor Overexpression on Anxiety and Memory after Early Life Stress in Female Mice

**DOI:** 10.3389/fnbeh.2015.00374

**Published:** 2016-01-26

**Authors:** Sofia Kanatsou, Judith P. Ter Horst, Anjanette P. Harris, Jonathan R. Seckl, Harmen J. Krugers, Marian Joëls

**Affiliations:** ^1^Department of Translational Neuroscience, Brain Center Rudolf Magnus, University Medical Center UtrechtUtrecht, Netherlands; ^2^Swammerdam Institute for Life Sciences, Center for Neuroscience, University of AmsterdamAmsterdam, Netherlands; ^3^Endocrinology Unit, Centre for Cardiovascular Science, Queen’s Medical Research Institute, The University of EdinburghEdinburgh, UK

**Keywords:** mineralocorticoid receptors, early life adversity, anxiety, fear, spatial memory

## Abstract

Early-life stress (ELS) is a risk factor for the development of psychopathology, particularly in women. Human studies have shown that certain haplotypes of *NR3C2*, encoding the mineralocorticoid receptor (MR), that result in gain of function, may protect against the consequences of stress exposure, including childhood trauma. Here, we tested the hypothesis that forebrain-specific overexpression of MR in female mice would ameliorate the effects of ELS on anxiety and memory in adulthood. We found that ELS increased anxiety, did not alter spatial discrimination and reduced contextual fear memory in adult female mice. Transgenic overexpression of MR did not alter anxiety but affected spatial memory performance and enhanced contextual fear memory formation. The effects of ELS on anxiety and contextual fear were not affected by transgenic overexpression of MR. Thus, MR overexpression in the forebrain does not represent a major resilience factor to early life adversity in female mice.

## Introduction

Stress experienced early in life may cause persisting changes in stress responsiveness, emotionality and cognitive performance, and increase the risk to develop psychopathologies, such as major depression, anxiety or personality disorders, later in life (De Bellis and Thomas, 2003; Cicchetti and Toth, 2005; Yasik et al., 2007; Green et al., 2010; Tottenham et al., 2010; Pechtel and Pizzagalli, 2011). Women are at higher risk of developing affective and cognitive disturbances under adverse life conditions than men (Lewinsohn et al., 1998; Kessler, 2003; Steiner et al., 2003; Wittchen and Jacobi, 2005; Bekker and van Mens-Verhulst, 2007).

Exposure to stressful events activates the hypothalamic-pituitary-adrenal (HPA) axis which increases the release of glucocorticoid hormones (Ulrich-Lai and Herman, 2009). These hormones—via binding to mineralocorticoid receptors (MRs) and glucocorticoid receptors (GR)—regulate neuronal excitability and behavioral adaptation via non-genomic as well as classical genomic mechanisms (reviewed in Joëls, 2011). Recent evidence suggests that an imbalance between MR and GR function, and especially reduced MR function, may be a critical risk factor to develop psychopathology. First, in a large population sample of aged individuals (men and women), polymorphisms of *NR3C2* encoding MR (MR-I180V single-nucleotide polymorphism (SNP)) associated with reduced MR function and an increased prevalence of depressive symptoms (Kuningas et al., 2007). Second, both in a healthy cohort of relatively young individuals and in a cohort enriched for affective disorders, an *NRC32* haplotype related to higher MR expression was found to be associated with a heightened dispositional optimism (Klok et al., 2011) and lower depression scores in the face of multiple life events, though significant effects were observed in women only (Klok et al., 2011; Vinkers et al., 2015; but see Hardeveld et al., 2015). In agreement, a recent study in humans revealed a beneficial effect of MR stimulation on cognition both in depressed patients and healthy (male and female) individuals (Otte et al., 2015).

One hypothesis to explain these studies is that enhanced MR levels moderate the effect of exposure to adverse life conditions, such as early life stress (ELS), at least in females. To test this we used a mouse model with transgenic overexpression of MR in the forebrain and explored the effects of ELS, induced by fragmented care of the dam towards her pups between postnatal days 2–9 (Rice et al., 2008). We specifically assessed behavioral tasks to explore cognitive performance in relevant spatial and emotional domains, in view of earlier findings in humans and rodents. Thus, children who spent the first part of life in institutionalized care showed decreased intellectual performance, language difficulties, poorer cognitive abilities and impaired psychomotor development compared to never institutionalized children (Rutter and O’Connor, 2004; Cohen et al., 2008; Loman et al., 2009; van den Dries et al., 2010). Furthermore, several studies have found significant impairments in verbal, visual, and global memory function in adulthood after ELS exposure (Bremner et al., 2003; Navalta et al., 2006; Bos et al., 2009). Similar results emerged from findings in animals indicating that ELS may enhance anxiety and fear memory formation (Aisa et al., 2007; Veenema et al., 2007; Oomen et al., 2011; Wang et al., 2012; Cohen et al., 2013) and impairs spatial learning and memory (Aisa et al., 2007, 2009; Ivy et al., 2010; Naninck et al., 2015; for review, see Loi et al., 2015). Thus, based on studies in female subjects, we were expecting that ELS impairs spatial memory formation (Champagne et al., 2008; Oomen et al., 2010) and enhances anxiety and fear memory formation (Champagne et al., 2008; Oomen et al., 2010).

## Materials and Methods

### Animals and Breeding Procedure

All mice were bred in-house by crossing male MR-tg mice with C57BL/6J females. One male animal was housed for 1 week with two female mice (Kanatsou et al., 2015a,b). The mouse line with forebrain MR overexpression (MR-tg) was generated by inserting the HA-tagged human MR cDNA using the CamKIIα promoter (Lai et al., 2007). Noticeable, the MR overexpression in this mouse model starts at PND15 (personal communication with JR Seckl). At the beginning of the third gestational week, pregnant females were single housed and observed daily for birth. All cages were covered with filter tops to prevent extra stress to the dams. When a litter was found, the day before was assigned as the day of birth (postnatal day 0 (PND0)). The dams with their litters were left undisturbed until PND2 and kept under standard housing conditions (12 h light/dark cycle, lights on at 8:00 a.m., humidity 40–60%, temperature 21.5–22.0°C) with unlimited access to food and water. The protocol was approved by the committee on Animal Health and Care from the University of Amsterdam, Netherlands (Permit Number: DED 291) and in accordance with the EC Council Directive of September 2010 (2010/63/EU). All efforts were made to minimize suffering.

### Chronic Early-Life Stress Paradigm

To induce ELS in pups, we used the limited nesting and bedding material (ELS) from PND2–PND9, as described previously (Rice et al., 2008; Naninck et al., 2015). Briefly, dams with their litters were weighed at PND2 and randomly assigned to the ELS or control condition. Litters were culled to six pups per litter including both genders. In total 24 litters were assigned to the control condition and 24 litters to the ELS condition. Control dams were provided with a normal amount of sawdust bedding and nesting material [one square piece of cotton nesting material (5 × 5 cm; Technilab-BMI, Someren, Netherlands)]. The ELS dams were provided with a reduced amount of sawdust bedding and nesting material [1/2 square piece of cotton nesting material (2.5 × 5 cm)] and a fine-gauge stainless steel mesh was placed 1 cm above the cage floor. Both control and ELS cages were left undisturbed until the cessation of the ELS regimen.

On PND9, dams and their litters were weighed and placed in standard cages, with sufficient bedding and nesting material until weaning at PND23. Upon weaning ear biopsies were collected for genotyping. At PND30 females were housed 3–4 per cage, each mouse from a different dam to avoid litter effects. All experimental mice were left undisturbed (except for cage cleaning once a week) until testing.

### Body Weights

To assess the effect of ELS on body weight, we measured the body weight of the female mice (*n* = 8–16 per group) at the following time points: PND23, PND39 and PND 120 (initiation of behavioral testing). The body weight records from PND23 and PND39 were obtained upon weaning and refreshing of the cages to avoid any unnecessary handling that could cause additional stress on top of the induction of the ELS paradigm.

### Behavior

All behavioral experiments were performed during the animals’ light phase (9:00 a.m. and 13:00 a.m.) by the same experimenter when the mice were 4 months old. Mice were subjected to a battery of behavioral tasks. Since activation of MRs depends on plasma corticosterone levels (de Kloet et al., 1999; Karst et al., 2005) MRs may play a different role in behavioral tasks which vary in the degree of salience. We therefore assessed the effects of chronic unpredictable stress and MR overexpression on behavior under low arousing and under high arousing experimental conditions, i.e., tasks that differ in the degree of activation of the HPA-axis.

Two different cohorts of animals were used for behavioral testing: the first batch for tasks with low arousal levels (open field, object in-context, object relocation) and the second batch for tasks with high induced arousal levels (contextual fear conditioning and extinction). In the low-arousal behavioral tests mice were examined in the following order: open field (part of object in-context, details explained below), object in-context and object relocation, with 5 days between each behavioral test. Behavioral performance of mice in the final task (object relocation) was comparable to that earlier observed in a pilot study in which mice were only subjected to the object relocation task (data not shown). This supports that exposure to the object-in-context task did not influence memory formation 5 days later in the object relocation task.

#### Open Field

Due to the experimental protocol of the object in-context, the first day—in these experiment-naïve mice—could be used to measure anxiety and exploratory behavior in an open field (day 1 context without objects, see below object in-context paradigm). Each mouse (*n* = 13–17 per group) was placed individually in the open field box (W × L × H; 33 cm × 54 cm × 37 cm) that contains bedding material on the bottom. All mice faced the same side of the open field and were allowed to explore the field for 10 min. After testing the mouse was removed from the open field and returned to its home cage. The walls of the open field were cleaned with 50% ethanol and new bedding material was added and mixed thoroughly with the old one. The analysis was performed using the Ethovision XT 6 (Noldus, Wageningen, Netherlands). We calculated the percentage of time spent in the center (reflecting anxiety levels) and the total distance mice travelled in the open field as an indicator of general exploratory behavior.

#### Object in-Context Recognition Memory

To examine the mice for context-dependent learning and memory object recognition we used the object in-context task, as described previously (Smith and Mizumori, 2006; Balderas et al., 2008; Spanswick and Sutherland, 2010; Kanatsou et al., 2015a,b). Briefly, mice (*n* = 13–17 per group) were tested for three subsequent days using boxes of identical measurements (W × L × H; 33 cm × 54 cm × 37 cm), either with or without visual cues on the walls and all contexts containing bedding material. On day 1 (open field box, with no wall cues and no objects) mice were allowed to explore for 10 min in order to habituate. The next day (day 2) mice were trained in two different boxes (context A: no wall cues, with a pair of same objects; context B: wall cues and a different pair of objects) for 10 min in each context. Objects were placed in opposite corners of the different side of the box and placed at a 15 cm distance from the corners of the box for both context A and context B. All objects were cleaned thoroughly between testing sessions. The retention time between testing in context A and context B was 1 min and mice were placed back in their home cage after training. On day 3, mice were tested for hippocampus-dependent object recognition memory in context B for 10 min, by replacing one of the pair of objects earlier seen in context B with an object that the mice had seen on day 2 in context A (i.e., the unfamiliar object for context B).

To assess place memory on day 3, we used the discrimination index (DI), calculating the time mice spent with the novel object compared to the total exploration time of both objects (t_novel_ /(t_novel +_ t_familiar_)) (Akkerman et al., 2012) in context B. Sniffing of the object was used as object-exploration behavior when the animal displayed such behavior within a distance of maximum 2 cm. Climbing on top of or watching the objects from a (close) distance was not considered as sniffing behavior.

#### Object Location

We used the object location test (OLT) to study spatial memory 5 days after testing the mice in object in-context. The protocol consisted of 3 days including habituation phase (day 1), training phase (day 2) and testing phase (day 3). For the arena, we used identical boxes (W × L × H; 23.5 cm × 31 cm × 27 cm) with no cues on the walls throughout the whole protocol while the bottom of the box was covered with bedding material. Mice (*n* = 13–17 per group) were allowed to explore in all phases for 5 min and returned to their home cages. On day 1, mice were habituated in the arena, followed 24 h later by a training session in which they were exposed to a pair of similar objects that were placed 12 cm from each other and 11 cm from the wall. On the testing day (day 3), 24 h post-training, one object was moved to a novel position and the mouse was allowed to explore the arena for 5 min. Fresh bedding material was added on top of the old and mixed thoroughly between sessions. As an index of spatial memory we used the DI as explained above.

#### Contextual Fear Conditioning and Extinction

Mice (*n* = 8–16 per group) were examined in the contextual fear conditioning task using a two-day protocol (Zhou et al., 2010). Briefly, on day 1, one mouse at a time was introduced in a chamber (W × L × H: 25 cm × 25 cm × 30 cm) that contained wall cues and a stainless steel grid floor connected to a shock generator and allowed to explore for 3 min. Immediately thereafter, mice received a single foot shock of 0.4 mA for 2 s and was removed 30 s later and placed back in its home cage. On day 2 (24 h post training), the mouse was tested for freezing behavior in the same chamber for 3 min. Extinction of conditioned fear was assessed by placing animals at day 3 and day 4 in the same chamber for 3 min, without giving a foot shock (as on day 2). The chamber was cleaned with 70% ethanol in-between each testing session. For consolidation of fear memory, we used the data on freezing behavior at day 2. Freezing behavior scored at day 3 and day 4 were used to measure extinction and compared to the data on day 2. To test whether testing over time reflects extinction or forgetting, we performed a pilot experiment (*n* = 8 control mice) in which testing of the mice at day 3 was omitted. In this pilot experiment (where we skipped day 3 of testing) we found that freezing level on day 4 was not reduced compared to day 2 (freezing levels on day 4: 28% ± 6 (mean ± SEM) and on day 2: 20% ± 1.6 (mean ± SEM). This argues against the interpretation of decreased freezing levels on day 4 (in animals tested on all days) as merely reflecting forgetting, but rather indicated extinction of fear response. In all experiments, freezing behavior was defined as no body movements except those related to breathing (Zhou et al., 2010) and was scored every 2 s. To assess fear memory, we calculated the total time spent freezing as a percentage of the total duration of the task.

### Determination of the Cycle Stage

The cycle stage of the females was determined by taking vaginal smears immediately after testing the mice in every single behavioral testing. The mouse was placed on top of the cage and using a smear loop we transferred cells on top of a water drop on a glass microscope slide. Slides dried overnight followed by Giemsa (Sigma) staining for 12 min. Subsequently, the cycle stage of the mice was defined using a light microscope.

### Statistical Analysis

Statistical analyses were performed using SPSS for Windows 17.0. For analysis of the body weight gain (to validate the ELS paradigm) we performed a two-tailed *t*-test. Repeated-measures ANOVA was used to analyze the absolute body weight and the extinction curves over the different days with within-subjects factor “testing day”, and “genotype” and “treatment” as fixed between-subject factors. Huynh-Feldt correction was used to correct for violations of sphericity. All other data were tested with a two-way ANOVA with ELS as the predicting factor and genotype as the moderating factor. Since we hypothesized that MR overexpression would moderate the effects of ELS, we were specifically interested in interaction effects of the two factors. Hence, only if the interaction effect was significant, a *post hoc* Fishers Least Significant Difference (LSD) test was performed; i.e., not in the case of main effects. To correct for multiple comparisons (four meaningful comparisons) the alpha was set to be significant at *p* ≤ 0.0125. To test the influence of the cycle stage in behavioral performance we used a General Linear Model analysis, including the cycle stage as a covariate. Data are presented as mean with standard error of the mean (SEM). Group sizes *(n)* used for the analysis are indicated in the sections above and in the figure legends.

## Results

### Body Weight

**Figure 1:**
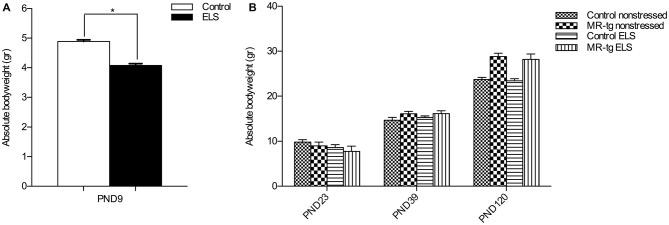
**Validation of early life stress (ELS) paradigm. (A)** Absolute body weight during the period of ELS (PND2–PND9). Female mice exposed to ELS (*n* = 108) had a significantly reduced bodyweight when compared to non-stressed female mice (*n* = 120). *Significant difference, *p* < 0.05, Student’s *t*-test. **(B)** Absolute body weights were recorded directly after weaning (PND23), at PND39 and at PND120 (initiation of behavioral testing). Data are expressed as mean + SEM, *n* = 8–16 mice per group.

To validate the effect of ELS, we recorded the bodyweight of the pups over the period PND2–PND9. Stressed female pups gained significantly less weight than control non-stressed female pups (*t*_(2,26)_ = 10.33, *p* < 0.001; Figure 1A). Subsequently, we measured body weight of the mice at three different time points (PND23, PND39, PND120). As expected body weight increased with age (repeated measures ANOVA, sphericity assumed correction: *F*_(2,88)_ = 752.013, *p* < 0.001, Figure 1B). In addition, there was a significant effect of genotype on weights over the three time points (repeated measures ANOVA, *F*_(2,88)_ = 21.573, *p* < 0.001), with MR-tg compared to wildtypes exhibiting a significantly greater increase in weights from PND23 to PND39 (*p* = 0.049) and from PND39 to PND120 (*p* < 0.001). There was no persisting effect of ELS on body weight (repeated measures ANOVA, *F*_(2,88)_ = 1.436, *p* = 0.243), and no interaction between genotype and ELS on bodyweight (repeated measures ANOVA, *F*_(2,88)_ = 0.052, *p* = 0.949).

### Behavior

Sub-analyses of the groups by cycle-stage did not reveal any significant influence of the cycle on the behavioral performance (data not shown); we acknowledge that this may be partly due to the relatively low number of females in some stages of the cycle. Therefore, we also grouped all stages and tested the impact of cycle stage on behavioral performance with a General Linear Model analysis, including the cycle stage as a covariate. Our results showed that significance of the results on behavioral performance did not change when the cycle stage was taking into account (anxiety: (*F*_(1,59)_ = 1.471, *p* = 0.230) object in-context (day 3): (*F*_(1,57)_ = 2.121, *p* = 0.151); object location (day 3): (*F*_(1,57)_ = 1.002, *p* = 0.322; fear conditioning (day 2): (*F*_(1,56)_ = 2.600, *p* < 0.113)).

#### Open Field

**Figure 2:**
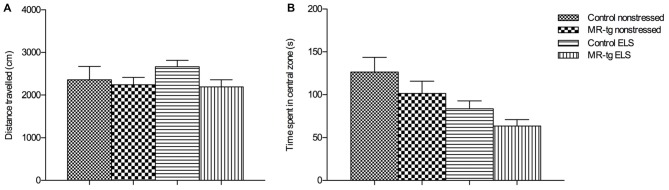
**Effect of ELS in the open field.** Bar graphs represent the results for: **(A)** exploratory behavior and locomotor activity after exposure to an open field, **(B)** time mice spent in the central zone of the open field, as a measure for anxiety-like behavior. Data are expressed as mean + SEM, *n* = 13–17 mice per group.

To examine exploratory behavior and locomotor activity we analyzed the total distance mice moved in an open field (Figure 2A). We found no main effect of genotype (*F*_(1,59)_ = 2.024, *p* = 0.160), nor an effect of ELS (*F*_(1,59)_ = 0.389, *p* = 0.536) or an interaction effect between ELS and genotype *F*_(1,59)_ = 0.774, *p* = 0.383) on the total distance mice moved, suggesting that all mice had the same exploratory and locomotor activity regardless whether they were exposed to the ELS paradigm or genetic modification.

To determine general anxiety, we analyzed the time mice spent in the central area of the open field. There was no interaction between ELS and genotype (*F*_(1,59)_ = 0.036, *p* = 0.851) nor a main effect of genotype (*F*_(1,59)_ = 2.944, *p* = 0.092) on the duration into the central zone but we found a main effect of ELS (*F*_(1,59)_ = 9.377, *p* < 0.003; Figure 2B), that is, ELS decreased the center time, in both genotypes.

#### Object In-Context Recognition Memory

**Figure 3:**
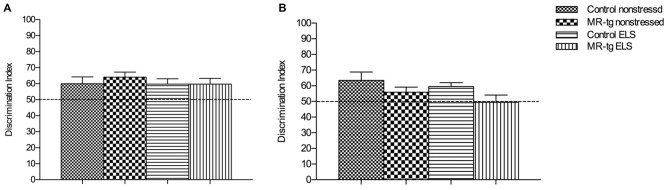
**Effect of ELS in spatial learning. (A)** In the object in-context recognition memory task, MR-tg and control mice showed comparable recognition memory after exposure to ELS. Data are expressed as mean ± SEM, *n* = 13–17 mice per group. **(B)** Effect of ELS in the object relocation. MR-tg and/or ELS caused no differences when mice were tested for spatial memory of the novel position of the object; no significant differences were observed in the discrimination index (DI) between the experimental groups. Data are expressed as mean + SEM, *n* = 13–17 mice per group.

We first used the object in-context test to determine the effect of ELS and increased forebrain MR expression on place learning and memory performance. Control (wildtype non-ELS) mice learned the task, since the DI was significantly higher than 50% (that is, chance level). Statistical analysis showed no main effect of genotype (*F*_(1,57)_ = 0.553, *p* = 0.460) or ELS (*F*_(1,57)_ = 0.343, *p* = 0.561) in memory performance. Furthermore, no interaction effect between ELS and genotype was observed (*F*_(1,57)_ = 0.280, *p* = 0.599, Figure 3A).

#### Object Location

To investigate the effect of ELS exposure on spatial memory in MR-tg and control mice, we used the object relocation task. Twenty-four hours post training we found that ELS did not affect the memory for the novel position of the object in the same context (*F*_(1,57)_ = 0.098, *p* = 0.183, Figure 3B). A main effect of genotype (*F*_(1,57)_ = 5.239, *p* = 0.026) was found, with MR-tg mice showing decreased spatial memory compared to control mice. No interaction effect between ELS and genotype (*F*_(1,57)_ = 0.280, *p* = 0.755) was found.

#### Contextual Fear Conditioning and Extinction

**Figure 4:**
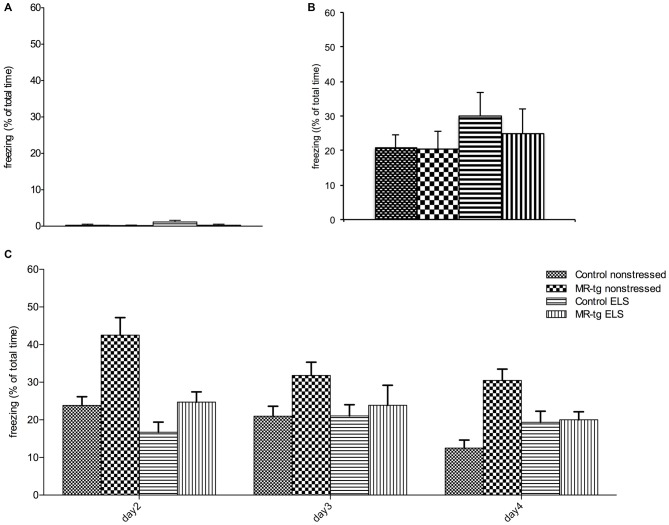
**Effect of ELS in consolidation of contextual fear conditioning and extinction.** Bar graphs represent the results for: **(A)** baseline freezing before the foot-shock on day 1, **(B)** time of freezing behavior immediately after the foot-shock on day 1, and **(C)** freezing during consolidation and recall (day 2) and extinction trials (day 2–day 4). **(A,B)** No differences in freezing behavior were observed between the four experimental groups during training. **(C)** During the retest on day 2 (consolidation), MR-tg non-stressed animals showed increased freezing levels compared to control non-stressed, and stressed animals exhibited a trend towards a decrease in freezing compared to non-stressed animals. None of the groups exhibited extinction. Data are expressed as mean + SEM, *n* = 8–16 mice per group.

We found neither an interaction effect between ELS and genotype (*F*_(1,56)_ = 1.513, *p* = 0.224, Figure 4A) nor an effect of ELS (*F*_(1,56)_ = 2.059, *p* = 0.157) or genotype (*F*_(1,56)_ = 2.059, *p* = 0.157) on freezing behavior in mice prior to the footshock. Mice from all groups also showed comparable freezing levels immediately after the foot-shock (Figure 4B).

Analysis of consolidation and retrieval of contextual fear (re-exposure to the same context on day 2, Figure 4C) revealed that MR-tg mice freeze more (main effect of genotype: (*F*_(1,56)_ = 13.627, *p* < 0.001)) while ELS decreased freezing (main effect of ELS: (*F*_(1,56)_ = 11.848, *p* < 0.001)), but the effect of ELS did not depend on genotype (interaction effect: (*F*_(1,56)_ = 2.192, *p* = 0.142)).

During extinction trials (day 2, day 3, day 4) a “within-subjects” effect analysis in freezing behavior showed a significant effect of day (repeated measures ANOVA, *F*_(2,104)_ = 8.590, *p* < 0.001, Figure 4C) and a significant interaction of day and genotype (repeated measures ANOVA, *F*_(2,104)_ = 6.634, *p* < 0.002). No significant interaction of day and treatment and genotype was found on extinction, therefore no *post hoc* comparisons have been performed.

## Discussion

We examined the role of MR overexpression in female mice that had experienced stress early in life (ELS), induced by exposure of the dam to limited nesting and bedding material. We report that ELS reduced the time that animals spend in the center of an open field but did not affect spatial discrimination learning. However, contextual fear memory was reduced, yet these animals did not show extinction of contextual fear. MR overexpression increased contextual fear but did not moderate behavioral changes after ELS.

### Body Weight

To examine the effects of ELS on learning and memory we raised dams and pups in an environment with limited nesting and bedding material between PND2–9. As reported before, the bodyweight of the pups between at PND9 was significantly decreased when compared to the pups that were not subjected to ELS (Rice et al., 2008; Naninck et al., 2015).

In the current model for MR overexpression, animals overexpress the MR gene starting from PND15. This implies that mice that were subjected to ELS did not have altered levels of the MR gene during the stress paradigm. Potential effects of MR overexpression can therefore be seen as a post-ELS interventional treatment, potentially reversing the earlier-induced ELS effects, although it cannot be excluded that some effects of ELS have a delayed onset which may be dampened by MR overexpression.

We observed that MR overexpressing females were heavier at 4 months of age when compared to control animals, a finding we observed before at the age of 3 months old, both in female (Kanatsou et al., 2015a) and male animals (Kanatsou et al., 2015b). A previous study showed that mice with MR deletion in the forebrain have a reduced bodyweight (Ter Horst et al., 2012). Our observation thus may supports previous evidence suggesting that forebrain MRs have a direct effect on total caloric intake and body weight gain, both under basal as well as increased corticosterone levels (Tempel and Leibowitz, 1994). However, this speculation needs to be further investigated in this model, as this is out of the scope of this study.

### Anxiety

In agreement with earlier observations (Wang et al., 2012) our data obtained from the open field showed a significant effect of ELS on anxiety with a trend towards increased anxiety in non-transgenic animals, as indicated by the reduced time that non-transgenic stressed animals spent in the center. Increased anxiety in the open field after early life adversity was earlier reported in several studies (e.g., Veenema et al., 2007; Tsuda and Ogawa, 2012) though not all studies examined female rodents (e.g., Rees et al., 2006; Wang et al., 2012). We did not find an effect of the MR transgene on anxiety as animals with MR overexpression spent a comparable amount of time in the center of the open field as controls. While earlier studies have reported that MR overexpression reduces anxiety in male animals (Lai et al., 2007; Rozeboom et al., 2007; Ferguson and Sapolsky, 2008), we have reported no effect of transgenic MR overexpression on anxiety in female mice and male mice in adulthood (Kanatsou et al., 2015a,b). Our results therefore suggest that MR overexpression does not affect anxiety and does not moderate the effects of ELS on anxiety in female animals, although it should be noted that this conclusion is based on a limited battery of tests.

### Learning and Memory Under Low-Arousal Conditions

It has previously been shown that the ELS paradigm that we used impairs memory formation in adult male mice (Rice et al., 2008; Wang et al., 2012; Naninck et al., 2015). However, little was known about how this particular model of ELS affects memory in female mice, which is a relevant question, since others report sex differences in susceptibility to ELS in rodents (Llorente et al., 2009; Oomen et al., 2009; reviewed in Loi et al., 2015). To further investigate this, we tested female mice for memory formation under low-arousing conditions (object in-context and object location) and in high-arousing tasks (contextual fear conditioning). ELS did not affect memory formation in the low-arousing memory tasks. Accordingly, Naninck et al. (2015) reported that ELS did not alter memory for object location in female mice. We furthermore found that discrimination learning is not affected by MR-overexpression, which is in agreement with earlier observations in MR-tg mice not exposed to ELS (Kanatsou et al., 2015a). Yet, Arp et al. (2014) reported that MR overexpression enhances spatial memory of females in a Barnes maze, which could indicate that effects of MR overexpression on memory performance may be task-dependent.

### Fear Memory and Extinction

Previous studies in male mice have shown that pharmacological blockade of the MR impairs contextual fear conditioning (Zhou et al., 2010). Recently we observed that MR overexpression in a transgenic mouse model enhances contextual fear memory in male mice (Kanatsou et al., 2015b). Here, we report that MR overexpression enhances contextual fear in (non-stressed) females. This may reflect enhanced consolidation which can result in increased perseverance (Harris et al., 2013) or altered MR-dependent alertness to act immediately when threatened. Whether this contributes to behavioral adaptation may depend on the context of the animals.

By contrast, ELS treatment decreased freezing behavior in female mice, suggesting that ELS hampers contextual fear memory formation. This agrees with several studies using rat models for early life adversity (Chocyk et al., 2014; Sun et al., 2014; Xiong et al., 2014), but other studies reported either no effect (Kosten et al., 2006; Diehl et al., 2007; Oomen et al., 2011) or even an enhanced contextual fear conditioning (Diehl et al., 2014). This suggests that various models of early life adversity or species differences may determine the effect of early life adversity on contextual fear. Transgenic overexpression of MRs did not alter freezing behavior in ELS animals, not supporting a moderating effect of MR on (the consequences of) ELS. One of the explanations could be that ELS potentially reduces MR expression (Vázquez et al., 1996) which could be compensated for by (subsequent) transgenic MR overexpression. However, other studies have not found altered MR expression levels after ELS (using the same model), either at PND28 or in adult age (Arp et al., under submission).

Furthermore, extinction learning was not affected by ELS but it did by MR overexpression. Possibly, extinction learning is hampered by MR overexpression, and/or MR overexpressing mice persevere in freezing behavior. The latter has been observed earlier (Harris et al., 2013; Gapp et al., 2014) and may reflect an impairment in behavioral flexibility or an alertness that is maintained in a potentially threatening context.

## Conclusion

The aim of this study was to determine whether—in female mice—the effects of ELS on anxiety and memory formation is moderated by transgenic overexpression of MRs. We observed that in female mice ELS increased anxiety and reduced contextual fear; these effects were not moderated by MR overexpression. Therefore, our data do not support that MR overexpression moderates the effects of early life adversity in female mice. Of note, MR overexpression in this particular model starts from PND15 onwards (Lai et al., 2007), i.e., 1 week after the period of limited bedding and nesting material, and as such can be regarded as a post-ELS intervention strategy. To what extent this is comparable to the situation of human MR haplotypes remains to be investigated.

## Author Contributions

Authors have made substantial contributions to the following: Conception and design of the study: SK, HJK, MJ; Interpretation of data: SK, HJK, MJ, JRS, APH; Acquisition of data: SK, JPTH; Analysis of data: SK; Drafting the article critically for important intellectual content: SK, HJK, MJ, JRS, APH, JPTH; Final approval of the version to be submitted: SK, HJK, MJ, JRS, APH, JTH; Agreement to be accountable for all aspects of the work in ensuring that questions related to the accuracy or integrity of any part of the work are appropriately investigated and resolved: SK, HJK, MJ, JRS, APH, JPTH.

## Conflict of Interest Statement

The authors declare that the research was conducted in the absence of any commercial or financial relationships that could be construed as a potential conflict of interest. The reviewer Shira Meir Drexler and handling Editor Oliver T. Wolf declared their shared affiliation, and the handling Editor states that the process nevertheless met the standards of a fair and objective review.
